# The role of disease history score in the association between depression history and current ME/CFS among US adults

**DOI:** 10.3389/fpubh.2026.1862602

**Published:** 2026-07-21

**Authors:** Song Mao, Liangxia Wu

**Affiliations:** Department of Pediatrics, Shanghai Sixth People’s Hospital Affiliated to Shanghai Jiao Tong University School of Medicine, Shanghai, China

**Keywords:** adult, depression, myalgic encephalomyelitis/chronic fatigue syndrome, past diseases, score

## Abstract

**Objective:**

To explore the role of disease history score in the association between depression history and current myalgic encephalomyelitis/chronic fatigue syndrome (ME/CFS) among US adults from 2021 to 2023.

**Patients and methods:**

This study derived data from the 2021 to 2023 US National Health Interview Survey (NHIS), a nationally representative cross-sectional survey. Current ME/CFS was defined using self-reported NHIS questions on a prior clinician diagnosis of ME/CFS and whether participants still had the condition.

**Results:**

A depression history was associated with higher odds of current ME/CFS (OR: 1.61, 95% CI: 1.12–2.32). A positive non-linear association between the disease history score and current ME/CFS was identified (OR: 2.49, 95% CI: 1.73–3.60). Above the threshold of −2.03, each unit increment in the disease history score was associated with 85% higher odds of current ME/CFS. No significant multiplicative or additive interaction was detected between the disease history score category and depression history in relation to current ME/CFS. In exploratory indirect association analyses, the disease history score and its category accounted for 47.2%/22.9 and 44.4%/24.1% of the association between depression history and current ME/CFS in the overall and female populations, respectively. The disease history score accounted for 59.2 and 30.5% of the depression-related association with current ME/CFS in the 50–64 years and 65 + years groups, respectively. Adding the disease history score to the model did not substantially improve reclassification or discriminatory performance for identifying current ME/CFS. The population attributable fraction (PAF) associated with depression history for current ME/CFS was higher than that associated with the disease history score category. Sensitivity analyses yielded similar results.

**Conclusion:**

The disease history score may partly explain the association between depression history and current ME/CFS status, especially among women and adults aged 50 years or older. Depression history was more strongly associated with current ME/CFS than increased multimorbidity burden. These findings support the potential relevance of depression history and multimorbidity burden in identifying adults with current ME/CFS, although longitudinal studies are needed to clarify temporal relationships.

## 1 Introduction

Myalgic encephalomyelitis/chronic fatigue syndrome (ME/CFS), a distinct chronic neurologic disorder, is characterized by over 6 months of fatigue that is unimproved by rest (1). ME/CFS has an average prevalence of 1.4%, with women being nearly 1.5- to 2-fold more frequently affected than men (2). ME/CFS presents with many symptoms, such as impaired memory, unrefreshing sleep, headache, and muscle pain (3). There is a significant overlap between ME/CFS and other chronic diseases (4). Due to the elusive etiology, diagnostic difficulty, and impaired quality of life associated with ME/CFS, identifying factors associated with current ME/CFS status is of great implication for clinical characterization and public health surveillance.

ME/CFS is frequently preceded by diverse infections, such as Epstein–Barr virus, human herpes virus 6, and COVID-19 (5). Autoimmune and inflammatory disorders also share some key pathogenic features with ME/CFS. For example, inflammatory bowel disease, herpes zoster, and psoriasis were associated with an increased risk of ME/CFS (6). Multiple biological pathways are involved in the development and progression of ME/CFS. Of note, adults with ME/CFS often experience multimorbidity burden, such as depression, which is one of the most common and costly illnesses in US, with a rising prevalence over the past three decades (7). Fatigue is one of the highly prevalent symptoms of depression (8). Meanwhile, depression is a well-recognized determinant of fatigue (9). The impact of depression on fatigue far exceeded other factors in patients with hemodialysis (10). Hence, an in-depth investigation of the role of depression in ME/CFS is of important clinical significance.

Accumulating evidence indicates that adults with depression and anxiety present an increased risk of ME/CFS, particularly among those taking antidepressants (11). Depression was a risk factor for fatigue in epilepsy patients (12). It was also observed that depression rates were significantly higher in ME/CFS adolescents than that in the general populations (13). ME/CFS with co-morbid psychopathology tend to be biased in thinking and fatigue symptoms (14). Current ME/CFS was operationally defined using NHIS questions on prior clinician-diagnosed ME/CFS and whether participants still had ME/CFS at the time of interview. Depression may be associated with current ME/CFS status by modulating fatigue severity and chronicity. However, the role of disease history in the association between depression history and current ME/CFS remains unclear. Hence, an in-depth investigation based on the impact of chronic disease history on the association between depression history and current ME/CFS is helpful for understanding the clinical profile of adults reporting current ME/CFS.

This study aimed to examine the role of disease history score in the association between depression history and current ME/CFS, as well as to quantify the respective association-based contributions of multimorbidity burden and depression history to current ME/CFS. The association between multimorbid disease history and current ME/CFS was investigated. Least absolute shrinkage and selection operator (LASSO) regression analysis was used to calculate the regression coefficients for each disease variable in relation to current ME/CFS.

The disease history score was the weighted sum of individual disease variables multiplied by their corresponding regression coefficients. Receiver operating characteristic (ROC) curve analysis was performed to evaluate the discriminatory performance of different models for identifying current ME/CFS. Piecewise linear regression was used to characterize the non-linear relationship between the disease history score and current ME/CFS. This study further assessed multiplicative and additive interactions between depression history and categorized disease history score in relation to current ME/CFS. Exploratory indirect association analyses were performed to assess whether the disease history score partly explained the association between depression history and current ME/CFS. Finally, the study calculated the population attributable fraction (PAF) for categorized disease history score and depression history in relation to current ME/CFS, while recognizing the cross-sectional and association-based nature of this estimate.

## 2 Materials and methods

### 2.1 Study design and population

This study used cross-sectional data from the 2021 to 2023 National Health Interview Survey (NHIS), which enrolled a representative sample of US households. NHIS is conducted by the National Center for Health Statistics of the Centers for Disease Control and Prevention. NHIS collected self-reported data on health topics. To obtain population-based estimates, each adult was assigned a weight, which was composed of a base sampling weight and adjustment of both screener and topical nonresponse, selection of a single adult, and various characteristics of adult in different states. Adults were defined as individuals aged 18 years or older. Due to the limit of data availability, ages more than 85 years were coded as 85 years in the data analysis.

Adults with a self-reported history of ME/CFS and available current ME/CFS status from the 2021 to 2023 NHIS database were included. Current ME/CFS was defined using two NHIS questions. Participants were first asked whether they had ever been told by a doctor or other health professional that they had CFS or ME. Those who answered “yes” were further asked whether they still had CFS or ME. Participants who answered “yes” to both questions were classified as having current ME/CFS. Therefore, current ME/CFS in this study reflects an NHIS-defined, self-reported current condition among adults reporting a prior clinician diagnosis, rather than an independently verified clinical diagnosis or a newly defined clinical subtype. Throughout this study, the term ME/CFS is used to refer to myalgic encephalomyelitis/chronic fatigue syndrome, including conditions historically described as chronic fatigue syndrome or ME/CFS in earlier studies.

Sociodemographic covariates included interview year, age, age group, sex, house region, district, race, educational level, BMI category, smoking status, and family poverty income ratio. The disease history variables included cancer, cancer count, epilepsy, anxiety disorder, dementia, arthritis, chronic obstructive pulmonary disease (COPD) emphysema or chronic bronchitis, diabetes, prediabetes, asthma, stroke, heart attack, angina, coronary heart disease, high cholesterol status, and hypertension. Individuals without complete covariate data were excluded. Participants were excluded if they did not meet the ME/CFS-history-based eligibility criteria, had unavailable current ME/CFS status, or had incomplete information on required disease history variables or covariates.

**Figure 1:**
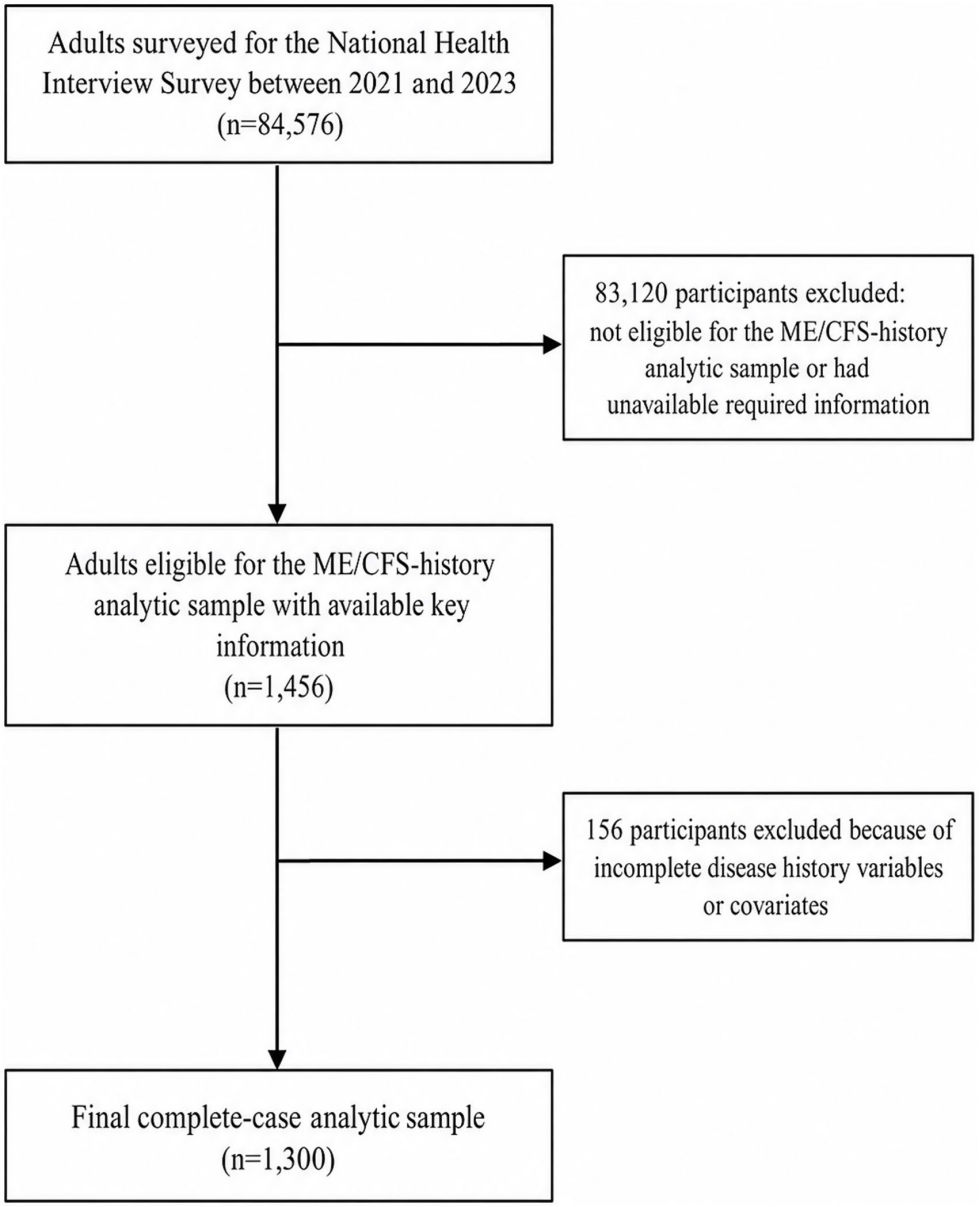
Selection and recruitment of participants in this study.

The final analytic sample was obtained by applying ME/CFS-history-based eligibility criteria and complete-case requirements. Participants were excluded if they did not have a self-reported history of ME/CFS, had unavailable current ME/CFS status, or had incomplete information on required disease history variables or covariates. These criteria were applied to define the target analytic population, the reduction from the full NHIS adult sample should not be interpreted as exclusion due to missing covariate data alone. After applying these eligibility and completeness criteria, 1,300 adults were included in the final analysis. The inclusion and exclusion process is presented in detail in Figure 1.

Ethical approval was not required for this study because it was a secondary analysis of publicly available, de-identified data from NHIS. No new data were collected, no participants were directly contacted, no intervention was performed, and no personally identifiable information was accessed or used. Hence, additional institutional ethical review and written informed consent were not required for this secondary analysis. The original NHIS data collection was conducted under the ethical review and informed-consent procedures of the data provider. This investigation followed the Strengthening the Reporting of observational studies in Epidemiology (SROBE) reporting guidelines.

### 2.2 Disease history score

The recruited disease history variables were cancer, cancer count, epilepsy, anxiety disorder, dementia, arthritis, COPD emphysema or chronic bronchitis, diabetes, prediabetes, asthma, stroke, heart attack, angina, coronary heart disease, high cholesterol status, and hypertension.

All disease history indicators were treated as binary variables and analyzed by the LASSO algorithm. LASSO is a regularization method that shrinks regression coefficients and selects relevant disease history variables, thereby reducing overfitting and improving the stability of the association model. In this study, the disease history score was constructed as a LASSO-derived, outcome-informed index of disease history associated with current ME/CFS. Therefore, it should not be interpreted as a conventional clinical multimorbidity scale independent of the outcome.

The continuous disease history score was created by summing the product of each binary disease history variable and its corresponding regression coefficient derived from the final LASSO model. The full scoring formula is as follows: disease history score = −0.53138 × *I* (anxiety = no) + (−0.56873) × *I* (arthritis = no) + (−0.16455) × *I* (COPD emphysema or chronic bronchitis = no) + (−0.02596) × *I* (dementia = no) + (−0.07391) × *I* (diabetes = no) + (−0.19227) × *I* (asthma = no) + 0.02416 × *I* (asthma = yes) + (−0.22831) × *I* (stroke = no) + (−0.22693) × *I* (angina = no) + (−0.02919) × *I* (coronary heart disease = no) + −0.06277 × *I* (hypertension = no). The condition of *I* is an indicator variable equal to 1 if the condition is met and 0 otherwise. The final variables retained in the LASSO model and their coefficients are provided in Table 1. Positive coefficients indicate a positive contribution to the calculated disease history score, whereas negative coefficients indicate a negative contribution.

**Table 1:** Coefficients of the LASSO model at the chosen lambda.

Comorbidities history		Coefficient
Anxiety	No	−0.53138
Arthritis	No	−0.56873
COPD emphysema or chronic bronchitis	No	−0.16455
Dementia	No	−0.02596
Diabetes	No	−0.07391
Asthma	No	−0.19227
Asthma	Yes	0.02416
Stroke	No	−0.22831
Angina	No	−0.22693
CHD	No	−0.02919
Hypertension	No	−0.06277

COPD, chronic obstructive pulmonary diseases; CHD, coronary heart disease; LASSO, least absolute shrinkage and selection operator. Coefficient represent the weights assigned to retained disease history categories at the selected lambda. A positive coefficient increases the disease history score, whereas a negative coefficient decreases the score. The absolute coefficient value reflects the relative contribution of each component to the LASSO-derived score. These coefficients should be interpreted as scoring weights rather than as causal effect estimates.

The score roughly followed the normal distribution with a median of −1.24, and a higher score indicates a higher LASSO-derived multimorbidity burden associated with current ME/CFS. Because the score was optimized in relation to current ME/CFS, the study interpreted it as an exploratory disease history index rather than as an independent causal construct.

### 2.3 Statistical analyses

The study calculates nationally representative estimates by including strata, cluster, and weight in the analyses. Baseline sociodemographic characteristics and disease history were summarized stratified by current ME/CFS status. This study presents weighted mean and proportions for continuous and categorical variables, respectively. Differences of variables among groups were assessed using survey-weighted linear regression test for continuous variables and survey-weighted χ2 tests for categorical variables. Logistic regression analyses to explore the associations between various disease histories and the odds of current ME/CFS are performed. Piecewise linear regression and threshold effect analyses were applied to evaluate the nonlinear relationships between the continuous disease history score, categorized score, and current ME/CFS.

ROC curve analysis was performed to assess the discriminatory performance of different models for identifying current ME/CFS. The net reclassification index (NRI) and integrated discrimination improvement (IDI) index were calculated to compare two models: sociodemographic characteristics + depression history versus sociodemographic characteristics + depression history + disease history score. This study further evaluates the multiplicative and additive interactions between depression history and categorized disease history score in relation to current ME/CFS. Exploratory indirect association analyses were performed to quantify the extent to which continuous and categorical disease history scores accounted for the association between depression history and current ME/CFS. Finally, the PAF was calculated for categorized disease history score and depression history to quantify their respective association-based contributions to current ME/CFS. Because of the cross-sectional design, these analyses were not interpreted as evidence of causal mediation or temporal sequencing.

In the sensitivity analyses, the robustness of the main results was tested by using propensity score matching (PSM)-processed data. The key analyses were repeated, including ROC-based discriminatory comparison, NRI and IDI evaluation, interaction test, exploratory indirect association analysis, and PAF estimation for disease history score and depression history. If available, an additional sensitivity analysis using a simple unweighted disease history count may be reported to examine whether the findings were robust to a non-LASSO multimorbidity burden measure.

The data analyses were performed using R software, version 4.2, provided by the R project for statistical computing, EmpowerStats (http://www.empowerstats.com, X&Y Solutions, Inc., Boston, MA) and Free Statistics analysis platform (Version 2.0, Beijing, China, http://www.clinicalscientists.cn/freestatistics). IRAP 2.2.0 (https://dceg.cancer.gov/tools/risk-assessment/irap) for PAF calculation. A two-sided *p* value less than 0.05 was considered statistically significant.

## 3 Results

### 3.1 Participants characteristics

Of 84,576 adult participants with available data from the 2021 to 2023 NHIS, 1,300 adults with complete sociodemographic characteristics, disease history variables, and current ME/CFS status were included in this study. The flow diagram of participants recruitment is shown in Figure 1. The primary reason for the reduced analytic sample was the restriction to adults reporting a prior history of ME/CFS and available current ME/CFS status. Participants with missing covariates were further excluded for complete-case analyses. The sample comprised 374 participants in 2021, 510 in 2022, and 416 in 2023.

Survey-weighted analyses showed no significant differences in year of interview, age, age group, sex, region, district, BMI category, smoking status, cancer history, cancer numbers, epilepsy history, dementia history, heart attack history, high cholesterol history, and hypertension history between participants with and without current ME/CFS. In contrast, significant differences were observed in race distribution, educational level, family poverty ratio, depression history, anxiety disorder history, arthritis history, COPD emphysema or chronic bronchitis history, diabetes history, prediabetes history, asthma history, stroke history, angina history, and coronary heart disease history between participants with and without current ME/CFS (Table 2).

**Table 2:** Baseline characteristics of adult participants with and without current ME/CFS (weighted).

Characteristics	Total (1,300)	Adults without current ME/CFS (263)	Adults with current ME/CFS (1037)	*p*
	Weighted estimate (95% CI)	Weighted estimate (95% CI)	Weighted estimate (95% CI)	
Year of interview				0.4197
2021	26.73 (24.05, 29.59)	23.08 (17.98, 29.12)	27.63 (24.60, 30.88)	
2022	43.34 (40.04, 46.70)	45.33 (38.57, 52.28)	42.84 (39.03, 46.74)	
2023	29.94 (26.88, 33.19)	31.58 (25.65, 38.18)	29.53 (26.08, 33.22)	
Age	54.10 (53.00, 55.21)	54.40 (52.27, 56.53)	54.03 (52.76, 55.30)	0.7685
Age group				0.8077
18 to 34 years	14.22 (11.69, 17.19)	13.58 (9.42, 19.18)	14.38 (11.55, 17.77)	
35 to 49 years	20.81 (18.18, 23.70)	22.64 (16.76, 29.84)	20.35 (17.56, 23.46)	
50 to 64 years	37.31 (34.26, 40.47)	34.89 (28.70, 41.65)	37.91 (34.44, 41.51)	
65 and 65 + years	27.66 (25.07, 30.41)	28.89 (23.41, 35.06)	27.35 (24.44, 30.48)	
Sex				0.2398
Female	67.16 (63.99, 70.18)	63.60 (56.88, 69.83)	68.05 (64.36, 71.53)	
Male	32.84 (29.82, 36.01)	36.40 (30.17, 43.12)	31.95 (28.47, 35.64)	
Household region				0.1021
Northeast	15.07 (12.88, 17.56)	19.69 (14.82, 25.67)	13.93 (11.27, 17.09)	
Midwest	21.82 (19.38, 24.47)	19.94 (15.26, 25.61)	22.28 (19.46, 25.39)	
South	42.58 (39.41, 45.81)	36.93 (30.36, 44.01)	4 3.99 (40.38, 47.66)	
West	20.53 (18.34, 22.90)	23.45 (17.97, 29.98)	19.80 (17.31, 22.55)	
District				0.2956
Nonmetropolitan	20.11 (17.47, 23.03)	16.77 (12.41, 22.27)	20.94 (17.86, 24.39)	
Medium and small metro	35.12 (31.09, 39.37)	34.08 (27.22, 41.67)	35.38 (31.12, 39.88)	
Large fringe metro	20.78 (17.55, 24.42)	20.42 (14.83, 27.43)	20.87 (17.34, 24.90)	
Large central metro	24.00 (20.78, 27.53)	28.73 (22.65, 35.69)	22.82 (19.46, 26.57)	
Race				0.0006
White only	83.44 (80.64, 85.90)	86.10 (80.57, 90.25)	82.78 (79.61, 85.55)	
African American only	9.58 (7.60, 12.00)	4.96 (2.82, 8.57)	10.72 (8.40, 13.60)	
Asian only	2.49 (1.64, 3.77)	4.12 (1.88, 8.81)	2.09 (1.27, 3.41)	
AIAN only	1.84 (1.11, 3.02)	1.15 (0.34, 3.79)	2.01 (1.18, 3.39)	
AIAN and any other group	1.55 (1.00, 2.41)	0.53 (0.12, 2.26)	1.81 (1.13, 2.86)	
Other single and multiple races	1.10 (0.62, 1.96)	3.13 (1.45, 6.65)	0.60 (0.24, 1.49)	
Educational level				0.0012
Grade 1 to 11	7.57 (6.05, 9.42)	8.25 (5.14, 12.97)	7.40 (5.68, 9.58)	
12th grade no diploma	2.51 (1.59, 3.94)	1.36 (0.27, 6.55)	2.80 (1.73, 4.50)	
GED or equivalent	4.67 (3.42, 6.35)	3.54 (1.60, 7.66)	4.95 (3.55, 6.87)	
High School Graduate	25.42 (22.51, 28.56)	20.34 (15.41, 26.35)	26.68 (23.39, 30.25)	
Some colleges no degree	21.41 (18.81, 24.27)	15.97 (11.73, 21.37)	22.77 (19.67, 26.19)	
Associate degree: occupational technical or vocational program	4.20 (3.18, 5.52)	3.96 (2.08, 7.42)	4.26 (3.12, 5.77)	
Associate degree: academic program	11.58 (9.71, 13.75)	13.00 (8.74, 18.90)	11.22 (9.24, 13.57)	
Bachelor’s degree	13.91 (11.89, 16.21)	16.80 (12.40, 22.36)	13.20 (11.08, 15.65)	
Master’s degree	6.28 (4.97, 7.89)	12.54 (8.82, 17.52)	4.72 (3.53, 6.30)	
Professional School or Doctoral degree	2.46 (1.69, 3.56)	4.26 (2.17, 8.20)	2.01 (1.29, 3.12)	
BMI category				0.1688
Underweight	2.60 (1.63, 4.11)	2.54 (1.16, 5.48)	2.61 (1.52, 4.44)	
Healthy weight	27.00 (24.15, 30.06)	31.43 (25.48, 38.07)	25.90 (22.73, 29.35)	
Overweight	26.72 (23.94, 29.70)	29.23 (23.06, 36.28)	26.10 (23.09, 29.35)	
Obese	43.68 (40.49, 46.91)	36.79 (30.02, 44.12)	45.39 (41.76, 49.06)	
Smoking status				0.8477
Never smoker	47.84 (44.49, 51.22)	49.18 (42.39, 55.99)	47.51 (43.77, 51.28)	
Former smoker	30.74 (27.79, 33.87)	31.52 (25.36, 38.41)	30.55 (27.26, 34.06)	
Current some day smoker	4.41 (3.30, 5.85)	4.44 (2.28, 8.47)	4.40 (3.19, 6.04)	
Current every day smoker	17.01 (14.72, 19.56)	14.87 (10.54, 20.56)	17.54 (14.98, 20.43)	
Family poverty ratio	3.10 (2.93, 3.26)	3.95 (3.53, 4.37)	2.89 (2.71, 3.06)	<0.0001
Ever had cancer				0.9299
No	83.39 (80.97, 85.56)	83.59 (78.12, 87.90)	83.35 (80.68, 85.71)	
Yes	16.61 (14.44, 19.03)	16.41 (12.10, 21.88)	16.65 (14.29, 19.32)	
Number of cancers				0.3443
No cancer	83.39 (80.97, 85.56)	83.59 (78.12, 87.90)	83.35 (80.68, 85.71)	
1 cancer	13.48 (11.48, 15.76)	14.86 (10.70, 20.28)	13.13 (10.95, 15.67)	
2 cancers	2.53 (1.83, 3.49)	1.55 (0.73, 3.26)	2.77 (1.93, 3.96)	
3 cancers	0.56 (0.28, 1.10)	0.00 (0.00, 0.00)	0.70 (0.36, 1.37)	
4 or more cancers	0.04 (0.01, 0.28)	0.00 (0.00, 0.00)	0.05 (0.01, 0.35)	
Ever had epilepsy				0.2309
No	91.43 (89.39, 93.11)	93.57 (89.14, 96.27)	90.90 (88.53, 92.81)	
Yes	8.57 (6.89, 10.61)	6.43 (3.73, 10.86)	9.10 (7.19, 11.47)	
Ever had depression				<0.0001
No	35.32 (32.44, 38.32)	49.97 (42.98, 56.95)	31.69 (28.52, 35.04)	
Yes	64.68 (61.68, 67.56)	50.03 (43.05, 57.02)	68.31 (64.96, 71.48)	
Ever had anxiety disorder				<0.0001
No	39.26 (36.27, 42.34)	53.90 (47.30, 60.37)	35.62 (32.34, 39.04)	
Yes	60.74 (57.66, 63.73)	46.10 (39.63, 52.70)	64.38 (60.96, 67.66)	
Ever had dementia				0.0736
No	96.14 (94.70, 97.19)	98.03 (95.40, 99.17)	95.66 (93.96, 96.90)	
Yes	3.86 (2.81, 5.30)	1.97 (0.83, 4.60)	4.34 (3.10, 6.04)	
Ever had arthritis				0.0003
No	35.33 (32.14, 38.66)	46.36 (39.51, 53.36)	32.59 (29.11, 36.27)	
Yes	64.67 (61.34, 67.86)	53.64 (46.64, 60.49)	67.41 (63.73, 70.89)	
Ever had COPD emphysema or chronic bronchitis				0.0104
No	78.55 (75.82, 81.05)	85.29 (79.67, 89.57)	76.88 (73.70, 79.78)	
Yes	21.45 (18.95, 24.18)	14.71 (10.43, 20.33)	23.12 (20.22, 26.30)	
Ever had diabetes				0.0277
No	80.46 (77.75, 82.91)	85.77 (80.62, 89.72)	79.14 (75.93, 82.02)	
Yes	19.54 (17.09, 22.25)	14.23 (10.28, 19.38)	20.86 (17.98, 24.07)	
Ever had prediabetes				0.0188
No	71.25 (68.21, 74.11)	77.62 (71.76, 82.57)	69.66 (66.17, 72.95)	
Yes	28.75 (25.89, 31.79)	22.38 (17.43, 28.24)	30.34 (27.05, 33.83)	
Ever had asthma				0.0009
No	65.30 (61.89, 68.56)	75.02 (68.85, 80.32)	62.88 (59.01, 66.60)	
Yes	34.70 (31.44, 38.11)	24.98 (19.68, 31.15)	37.12 (33.40, 40.99)	
Ever had a stroke				0.0152
No	91.64 (89.92, 93.09)	95.29 (92.08, 97.24)	90.73 (88.67, 92.44)	
Yes	8.36 (6.91, 10.08)	4.71 (2.76, 7.92)	9.27 (7.56, 11.33)	
Ever had a heart attack				0.4412
No	92.15 (90.37, 93.62)	93.51 (88.81, 96.32)	91.81 (89.79, 93.45)	
Yes	7.85 (6.38, 9.63)	6.49 (3.68, 11.19)	8.19 (6.55, 10.21)	
Ever had angina				0.0223
No	91.42 (89.55, 92.97)	95.23 (91.51, 97.36)	90.47 (88.27, 92.29)	
Yes	8.58 (7.03, 10.45)	4.77 (2.64, 8.49)	9.53 (7.71, 11.73)	
Ever had coronary heart disease				0.0443
No	85.73 (83.46, 87.74)	89.69 (85.27, 92.89)	84.75 (82.12, 87.06)	
Yes	14.27 (12.26, 16.54)	10.31 (7.11, 14.73)	15.25 (12.94, 17.88)	
Ever had high cholesterol				0.4640
No	53.94 (50.62, 57.24)	56.28 (49.59, 62.74)	53.37 (49.47, 57.22)	
Yes	46.06 (42.76, 49.38)	43.72 (37.26, 50.41)	46.63 (42.78, 50.53)	
Ever had hypertension				0.1336
No	48.37 (45.04, 51.71)	53.41 (46.23, 60.46)	47.11 (43.29, 50.97)	
Yes	51.63 (48.29, 54.96)	46.59 (39.54, 53.77)	52.89 (49.03, 56.71)	

ME/CFS, myalgic encephalomyelitis/chronic fatigue syndrome; AIAN, American Indian/Alaska Native; GED, general educational development, variables: survey-weighted percentage (95% CI), survey-weighted Chi-square test.

### 3.2 Association between disease history and current myalgic encephalomyelitis/chronic fatigue syndrome

**Figure 2:**
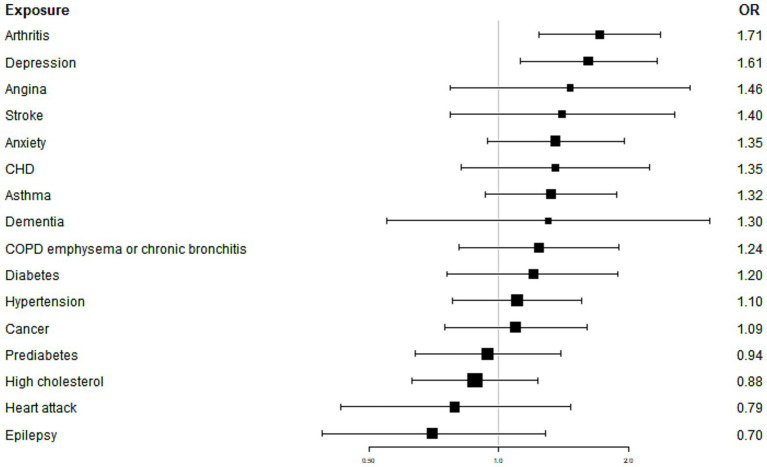
Odds ratios of current myalgic encephalomyelitis/chronic fatigue syndrome in relation to various disorders.

Arthritis history was associated with 71% higher odds of current ME/CFS than no arthritis history in model 2 (OR: 1.71, 95% CI: 1.24–2.36, *p* = 0.0011, Table 3). Depression history was associated with 61% higher odds of current ME/CFS than no depression history in model 2 (OR: 1.61, 95% CI: 1.12–2.32, *p* = 0.0101, Table 3). No significant associations between histories of diabetes, prediabetes, angina, anxiety, asthma, cancer, CHD, high cholesterol status, COPD emphysema or chronic bronchitis, dementia, epilepsy, hypertension, heart attack, and stroke and current ME/CFS were identified (Table 3). Figure 2 showed the ORs of current ME/CFS in exposure to various diseases.

**Table 3:** Association between disorder history with current ME/CFS in adults.

Exposure	Crude	Model I	Model II
Diabetes	1.43 (1.00, 2.06) 0.0511	1.28 (0.87, 1.89) 0.2158	1.20 (0.76, 1.88) 0.4371
Prediabetes	1.26 (0.93, 1.71) 0.1354	1.12 (0.80, 1.57) 0.4948	0.94 (0.64, 1.39) 0.7700
Angina	2.07 (1.16, 3.67) 0.0131	1.94 (1.07, 3.51) 0.0295	1.46 (0.77, 2.76) 0.2505
Anxiety	1.96 (1.49, 2.57) < 0.0001	1.85 (1.37, 2.50) < 0.0001	1.35 (0.94, 1.95) 0.1044
Arthritis	2.13 (1.61, 2.81) < 0.0001	1.90 (1.39, 2.60) < 0.0001	1.71 (1.24, 2.36) 0.0011
Asthma	1.67 (1.23, 2.27) 0.0011	1.52 (1.10, 2.10) 0.0113	1.32 (0.93, 1.87) 0.1214
Cancer	1.02 (0.73, 1.44) 0.8908	1.11 (0.77, 1.61) 0.5654	1.09 (0.75, 1.60) 0.6480
CHD	1.48 (1.00, 2.21) 0.0523	1.53 (0.99, 2.37) 0.0563	1.35 (0.82, 2.23) 0.2445
High cholesterol	1.15 (0.88, 1.51) 0.2998	1.07 (0.79, 1.44) 0.6685	0.88 (0.63, 1.23) 0.4544
COPD emphysema or chronic bronchitis	1.81 (1.26, 2.61) 0.0015	1.57 (1.06, 2.32) 0.0236	1.24 (0.81, 1.89) 0.3274
Dementia	1.77 (0.79, 3.97) 0.1623	1.81 (0.78, 4.17) 0.1674	1.30 (0.55, 3.07) 0.5467
Epilepsy	1.18 (0.68, 2.03) 0.5564	0.97 (0.55, 1.71) 0.9224	0.70 (0.39, 1.28) 0.2511
Hypertension	1.36 (1.04, 1.79) 0.0251	1.19 (0.86, 1.63) 0.2918	1.10 (0.78, 1.55) 0.5832
Depression	2.13 (1.62, 2.81) < 0.0001	2.02 (1.50, 2.71) < 0.0001	1.61 (1.12, 2.32) 0.0101
Heart attack	1.40 (0.83, 2.37) 0.2024	1.21 (0.70, 2.09) 0.4950	0.79 (0.43, 1.47) 0.4560
Stroke	1.91 (1.11, 3.28) 0.0196	1.70 (0.96, 2.98) 0.0671	1.40 (0.77, 2.55) 0.2672

ME/CFS, myalgic encephalomyelitis/chronic fatigue syndrome; CHD, coronary heart disease Indian/Alaska Native; COPD, chronic obstructive pulmonary diseases, OR (95% CI) P. Model I: adjust for age, BMI category, educational level, poverty ratio, race, region, sex, smoking status, and district, Model II (exclusion of dependent variable): adjust for age, BMI category, educational level, poverty ratio, race, region, sex, smoking status, and district, and prediabetes, angina, anxiety, arthritis, asthma, cancer, CHD, COPD emphysema or chronic bronchitis, dementia, depression, diabetes, epilepsy, hypertension, heart attack, stroke, and high cholesterol.

### 3.3 Nonlinear association between disease history score and current myalgic encephalomyelitis/chronic fatigue syndrome

**Figure 3:**
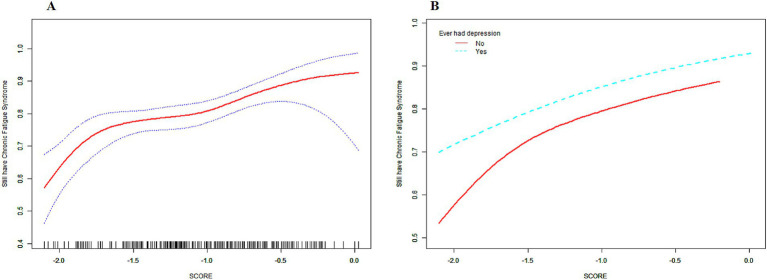
Nonlinear relationship between disease history score and current myalgic encephalomyelitis/chronic fatigue syndrome: **(A)** Total participants, **(B)** subgroup analysis in terms of depression history.

Threshold effect analysis showed a significant positive relationship between disease history score and current ME/CFS in model II (OR: 2.49, 95% CI: 1.73–3.60, *p* < 0.0001). The threshold point of disease history score in relation to current ME/CFS was −2.03 in all models (crude, models I and II, Table 4). Above the threshold of −2.03, each unit increment in the disease history score was associated with 85% higher odds of current ME/CFS in model II (Table 4). Figure 3 showed the nonlinear relationship between disease history score and current ME/CFS.

**Table 4:** Threshold effect analysis of disease history score and current ME/CFS.

Outcome	Crude	Model I	Model II
Total effect	3.48 (2.55, 4.75) < 0.0001	2.94 (2.10, 4.13) < 0.0001	2.49 (1.73, 3.60) < 0.0001
TP (95% CI)	−2.03 (−2.08, −1.72)	−2.03 (−2.08, −1.66)	−2.03 (−2.08, −1.71)
OR (95% CI) P < TP	NA	NA	NA
OR (95% CI) P > TP	2.43 (1.70, 3.46) < 0.0001	2.17 (1.48, 3.16) < 0.0001	1.85 (1.24, 2.77) 0.0026

ME/CFS, myalgic encephalomyelitis/chronic fatigue syndromel; OR, odds ratio; TP, threshold point; Crude, no adjusted factors, Model I: adjust for age, BMI category, educational level, poverty ratio, race, region, sex, smoking status, and district, Model II: adjust for age, BMI category, educational level, poverty ratio, race, region, sex, smoking status, district and depression history, NA, not applicable.

### 3.4 Interaction between disease history score and depression history in relation to current myalgic encephalomyelitis/chronic fatigue syndrome

**Figure 4:**
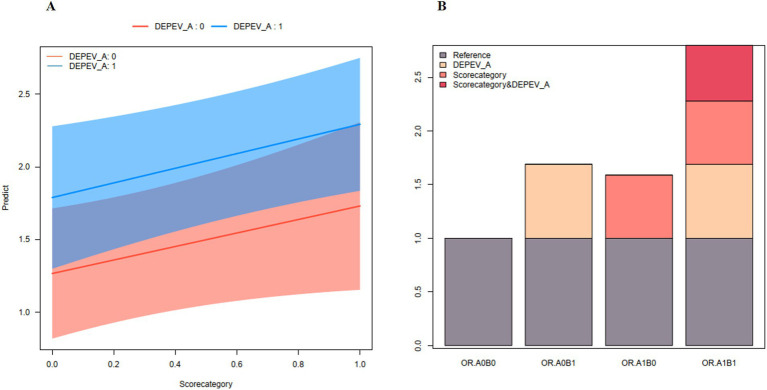
Interaction between disease history score and depression history in relation to current myalgic encephalomyelitis/chronic fatigue syndrome: **(A)** Multiplicative interaction analysis, **(B)** additive interaction analysis, DEPEV_A:0: no depression history, DEPEV_A:1: depression history, A0B0: lower score without depression history, A0B1: lower score with depression history, A1B0: higher score without depression history, A1B1: higher score with depression history.

Interaction analysis showed that the values of multiplicative scale, relative excess risk due to interaction (RERI), attributable proportion (AP), and synergy index (SI) in all models (crude and model I) were not significant. The 95% CIs of RERI and AP included 0, and the 95% CIs of multiplicative scale and SI included 1. These findings indicated that no significant multiplicative or additive interaction between disease history score category and depression history existed in relation to current ME/CFS (Table 5). The disease history score category was defined as lower score group versus higher score group, with lower score < median value of disease history score and higher score ≥ median value of disease history score. Figure 4 showed the multiplicative and additive interaction patterns between disease history score and depression history in relation to current ME/CFS.

**Table 5:** Interaction between disease history score category and depression history in relation to current ME/CFS.

Group	Crude OR (95% CI)	Model I OR (95% CI)
Lower disease history score (< median)		
Without depression	Reference	Reference
With depression	1.68 (1.17, 2.40)	1.66 (1.13, 2.44)
Higher disease history score (≥ median)		
without depression	1.83 (1.13, 2.97)	1.53 (0.91, 2.55)
with depression	3.26 (2.31, 4.61)	2.76 (1.91, 4.00)
Multiplicative scale	1.06 (0.57, 1.98)	1.04 (0.55, 1.97)
RERI	0.76 (−0.5, 2.02)	0.52 (−0.63, 1.67)
AP	0.23 (−0.12, 0.58)	0.18 (−0.2, 0.57)
SI	1.5 (0.72, 3.16)	1.4 (0.61, 3.2)

ME/CFS: myalgic encephalomyelitis/chronic fatigue syndrome, OR: odds ratio, RERI: relative excess risk due to interaction, AP: attributable proportion due to interaction, SI: synergy index, Crude: no adjust Model I: adjust for age, BMI category, educational level, poverty ratio, race, region, sex, smoking status, and district.

### 3.5 Exploratory indirect association analysis of disease history score in the depression–current myalgic encephalomyelitis/chronic fatigue syndrome association

The continuous disease history score and categorized score accounted for 47.2 and 22.9% of the association between depression history and current ME/CFS in the whole participants, respectively (model I, Table 6). The continuous disease history score and categorized score accounted for 44.4 and 24.1% of the association between depression history and current ME/CFS in females, respectively (model I, Table 6), whereas no significant indirect associations were detected in males (model I, Table 6). Subgroup analyses in terms of age showed no significant indirect association in the 18–34 years and 35–49 years groups. For the 50–64 years and 65 years and over groups, the continuous disease history score accounted for 59.2 and 30.5% of the depression-related association with current ME/CFS, respectively (model I, Table 6). Given the cross-sectional design, these findings should be interpreted as exploratory indirect associations rather than evidence of causal mediation.

**Table 6:** Exploratory indirect association analysis of disease history score in the depression-current ME/CFS association

Exposure	Crude β/OR (95% CI)	*p*	Model I OR (95% CI) β/OR (95% CI)	*p*
Score				
Total effect	0.120 (0.075, 0.172)	<0.0001	0.107 (0.062, 0.162)	<0.0001
Direct effect	0.058 (0.012, 0.110)	0.018	0.0564 (0.011, 0.112)	0.018
Mediation effect	0.062 (0.040, 0.089)	<0.0001	0.050 (0.028, 0.077)	<0.0001
Proportion mediated effect	0.519 (0.306, 0.855)	<0.0001	0.472 (0.234, 0.847)	<0.0001
Score category				
Total effect	0.126 (0.075, 0.170)	<0.0001	0.112 (0.0618, 0.158)	<0.0001
Direct effect	0.090 (0.036, 0.137)	<0.0001	0.086 (0.037, 0.136)	<0.0001
Mediation effect	0.036 (0.018, 0.057)		0.0256 (0.006, 0.044)	0.010
Proportion mediated effect	0.287 (0.135, 0.537)	<0.0001	0.229 (0.058, 0.482)	0.010
Female				
Score				
Total effect	0.128 (0.071, 0.189)	<0.0001	0.120 (0.066, 0.179)	<0.0001
Direct effect	0.067 (0.012, 0.135)	0.0240	0.067 (0.012, 0.126)	0.026
Mediation effect	0.061 (0.028, 0.091)	<0.0001	0.053 (0.030, 0.082)	<0.0001
Proportion mediated effect	0.475 (0.202, 0.849)	<0.0001	0.444 (0.226, 0.838)	<0.0001
Score category				
Total effect	0.133 (0.081, 0.197)	<0.0001	0.119 (0.066, 0.179)	<0.0001
Direct effect	0.277 (0.129, 0.566)	<0.0001	0.091 (0.036, 0.154)	0.002
Mediation effect	0.037 (0.018, 0.066)	<0.0001	0.0287 (0.012, 0.053)	<0.0001
Proportion mediated effect	0.277 (0.129, 0.566)	<0.0001	0.241 (0.0858, 0.539)	<0.0001
Male				
Score				
Total effect	0.097 (0.012, 0.232)	0.024	0.082 (−0.0116, 0.168)	0.0840
Direct effect	0.040 (−0.045, 0.175)	0.324	0.0467 (−0.0509, 0.1396)	0.3140
Mediation effect	0.057 (0.018, 0.109)	0.016	0.0356 (−0.006, 0.077)	0.0820
Proportion mediated effect	0.585 (0.053, 2.340)	0.040	0.432 (−1.368, 2.568)	0.162
Score category				
Total effect	0.098 (0.012, 0.178)	0.026	0.083 (−0.0119, 0.169)	0.0820
Direct effect	0.072 (−0.016, 0.158)	0.106	0.0729 (−0.0227, 0.164)	0.1180
Mediation effect	0.0269 (−0.009, 0.059)	0.134	0.0101 (−0.0286, 0.0433)	0.616
Proportion mediated effect	0.273 (−0.144, 1.220)	0.156	0.122 (−0.795, 1.230)	0.638
18–34 years				
Score				
Total effect	−0.0949 (−0.266, 0.111)	0.2960	0.033 (−0.164, 0.270)	0.684
Direct effect	−0.2243 (−0.3869, −0.0205)	0.0320	−0.057 (−0.2366, 0.1941)	0.628
Mediation effect	0.1295 (0.01938, 0.2638)	0.0020	0.090 (0.006, 0.2099)	0.038
Proportion mediated effect	−1.3649 (−19.346, 9.874)	0.2980	2.73 (−7.930, 13.514)	0.682
Score category				
Total effect	−0.048 (−0.225, 0.145)	0.514	0.055 (−0.1443, 0.2923)	0.53
Direct effect	−0.076 (−0.245, 0.106)	0.358	0.0226 (−0.169, 0.273)	0.730
Mediation effect	0.028 (−0.011, 0.0706)	0.156	0.0326 (−0.018, 0.0975)	0.254
Proportion mediated effect	−0.584 (−4.603, 3.396)	0.586	0.591 (−3.972, 4.480)	0.632
35–49 years				
Score				
Total effect	0.179 (0.036, 0.334)	0.004	0.104 (−0.020, 0.223)	0.098
Direct effect	0.035 (−0.111, 0.1636)	0.7140	0.034 (−0.087, 0.173)	0.614
Mediation effect	0.1435 (0.0804, 0.248)	<0.0001	0.070 (0.018, 0.128)	0.010
Proportion mediated effect	0.803 (0.421, 3.137)	0.004	0.673 (−3.000, 4.046)	0.108
Score category				
Total effect	0.186 (0.060, 0.315)	0.006	0.106 (−0.012, 0.228)	0.088
Direct effect	0.105 (−0.015, 0.236)	0.076	0.067 (−0.051, 0.204)	0.276
Mediation effect	0.0807 (0.0253, 0.131)	0.004	0.0384 (−0.001, 0.085)	0.058
Proportion mediated effect	0.434 (0.127, 1.185)	0.010	0.363 (−1.620, 2.187)	0.142
50–64 years				
Score				
Total effect	0.141 (0.046, 0.219)	<0.0001	0.0929 (0.0187, 0.1708)	0.014
Direct effect	0.087 (−0.003, 0.192)	0.062	0.038 (−0.046, 0.127)	0.3620
Mediation effect	0.054 (−0.020, 0.095)	0.182	0.055 (0.0118, 0.1012)	0.010
Proportion mediated effect	0.383 (−0.213, 1.056)	0.182	0.592 (0.0989, 2.509)	0.024
Score category				
Total effect	0.146 (0.0709, 0.223)	<0.0001	0.0896 (0.0174, 0.171)	0.010
Direct effect	0.112 (0.031, 0.204)	0.012	0.064 (−0.0187, 0.154)	0.110
Mediation effect	0.034 (−0.004, 0.0724)	0.092	0.0257 (−0.0084, 0.065)	0.138
Proportion mediated effect	0.231 (−0.032, 0.642)	0.092	0.287 (−0.131, 1.411)	0.148
65 years and over				
Score				
Total effect	0.129 (0.0517, 0.205)	<0.0001	0.124 (0.051, 0.196)	<0.0001
Direct effect	0.088 (0.0108, 0.172)	0.028	0.086 (0.0109, 0.161)	0.032
Mediation effect	0.041(0.0089, 0.069)	0.010	0.038 (0.013, 0.067)	0.006
Proportion mediated effect	0.318 (0.0713, 0.843)	0.010	0.305 (0.097, 0.818)	0.006
Score category				
Total effect	0.129 (0.055, 0.204)	<0.0001	0.127 (0.050, 0.198)	<0.0001
Direct effect	0.103 (0.022, 0.182)	0.014	0.109 (0.0289, 0.179)	0.01
Mediation effect	0.026 (−0.002, 0.057)	0.066	0.0183 (−0.008, 0.0470)	0.222
Proportion mediated effect	0.203 (−0.015, 0.652)	0.066	0.144 (−0.070, 0.509)	0.222

ME/CFS: myalgic encephalomyelitis/chronic fatigue syndrome, OR: odds ratio, Crude: no adjust Model I: adjust for age, BMI category, educational level, poverty ratio, race, region, sex, smoking status, and district.

### 3.6 Discriminatory performance of different models for current myalgic encephalomyelitis/chronic fatigue syndrome

**Figure 5:**
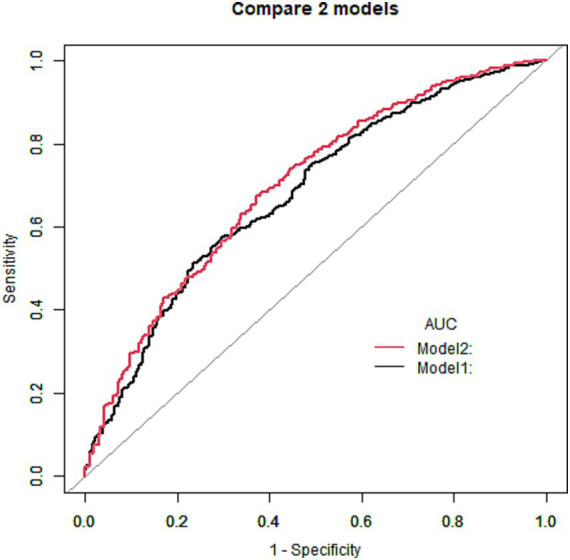
Comparison of AUC between two models for current myalgic encephalomyelitis/chronic fatigue syndrome (model 1: Social, demographic + Dep model, model 2: Social, demographic + Dep + Score Model).

The sociodemographic characteristics + depression model showed a significantly higher area under the curve (AUC) (0.6779 vs. 0.6578) compared with the sociodemographic characteristics only model (Table 7). Further inclusion of the disease history score significantly improved the AUC (0.6991 vs. 0.6779) (Table 7; Figure 5). Nevertheless, no significant differences in the NRI and IDI were observed between the sociodemographic characteristics + depression + disease history score model, and the sociodemographic characteristics + depression model. These findings suggest that although adding the disease history score slightly improved model discrimination as reflected by AUC, it did not provide a significant incremental improvement in risk reclassification or overall discrimination improvement according to NRI and IDI. Therefore, the incremental predictive value of the disease history score should not be overinterpreted (Table 8).

**Table 7:** Discriminatory performance of different models for current ME/CFS.

Models	AUC	95% CI	Accuracy	Sensitivity	Specificity	PPV	NPV	*p*
^**1**^Social, demographic	0.6578	0.6233–0.6910	0.6638	0.6933	0.5475	0.8580	0.3117	
^**2**^Social, demographic + Dep	0.6779	0.6440–0.7126	0.6023	0.5767	0.7034	0.8846	0.2965	0.0489 2 vs. 1
^**3**^Social, demographic + Dep + Score	0.6991	0.6668–0.7329	0.6692	0.6818	0.6198	0.8761	0.3306	0.0117 3 vs. 2

^1^Model 1 included social and demographic variables. ^2^Model 2 included social and demographic variables and depression history. ^3^Model 3 included social and demographic variables, depression history, and the disease history score ROC, receiver operating curve; ME/CFS, myalgic encephalomyelitis/chronic fatigue syndrome; Dep, depression; PPV, positive predictive value; NPV, negative predictive value.

**Table 8:** NRI and IDI for the analysis of different models for current ME/CFS (Social, demographic + Dep vs. Social, demographic + Dep + Score).

Index	Estimate	*p*-value	95% lower	95% upper
NRI	0.0215	0.4025	−0.0288	0.0717
IDI	0.0215	0.4032	−0.0289	0.0718

ME/CFS, myalgic encephalomyelitis/chronic fatigue syndrome; Dep, depression; NRI, net reclassification index; IDI, integrated discrimination index.

### 3.7 PAF of disease history score and depression history for current myalgic encephalomyelitis/chronic fatigue syndrome

**Figure 6:**
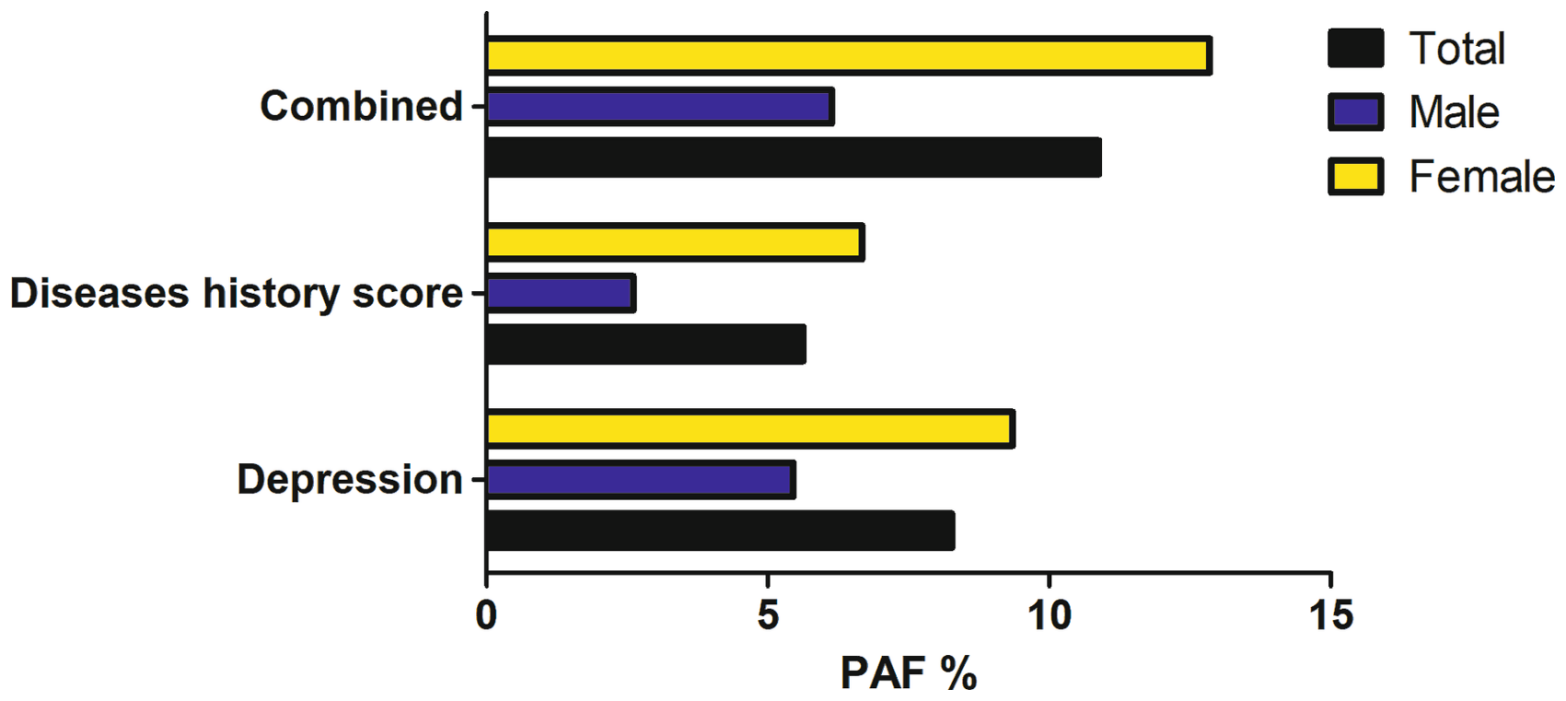
PAF of disease history score and depression history for current myalgic encephalomyelitis/chronic fatigue syndrome.

Table 9 and Figure 6 presented the PAF of categorized disease history score and depression history for current ME/CFS. Depression history in females had a PAF of 9.34% for current ME/CFS. The combination of elevated disease history score and depression history showed the highest PAF of 12.85% for current ME/CFS in females. Depression history contributed more to current ME/CFS than higher disease history score. Because the data were cross-sectional, these PAF estimates should be interpreted as association-based estimates rather than causal preventable fractions.

**Table 9:** PAF of disease history score category and depression history for current ME/CFS.

Exposure	PAF, % (95% CI)
Total	
Depression	8.27 (4.58–11.96)
Diseases history score category	5.63 (2.94–8.31)
Diseases history score category + Depression	10.88 (6.52–15.23)
Female	
Depression	9.34 (4.72–13.97)
Diseases history score category	6.67 (3.29–10.04)
Diseases history score category + Depression	12.85 (7.21–18.48)
Male	
Depression	5.45 (−0.54–11.43)
Diseases history score category	2.61 (−1.96–7.17)
Diseases history score category + Depression	6.13 (−0.60–12.85)

PAF, Population attributable fraction; OR, odds ratio; CI, confidence intervals. Adjust for district, race, smoking, BMI category, educational level, region, sex, age category, and poverty ratio category.

### 3.8 Sensitivity analyses

To exclude the influence of various sociodemographic variables, PSM of sociodemographic variables between non-depression and depression history participants was performed, which can better show the difference of current ME/CFS between non-depression and depression history participants. PSM yielded a matched sample of 874 adults (437 depression vs. 437 non-depression). The SMDs of all sociodemographic variables between non-depression and depression participants were all less than 0.1, which showed a good match of covariates between non-depression and depression participants (Supplementary Table 1). Fisher’s exact test showed that depression history was significantly associated with an increased proportion of current ME/CFS (*p* < 0.0001). Logistic regression analysis showed that depression history was associated with higher odds of current ME/CFS (OR: 2.03, 95% CI: 1.45–2.85, *p* < 0.0001).

**Figure 7:**
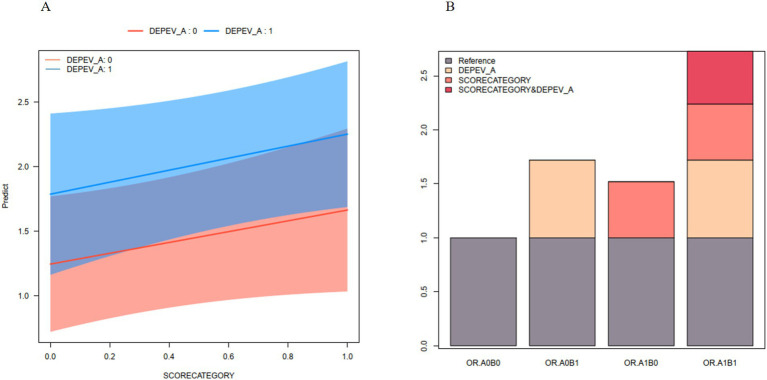
Interaction between disease history score and depression history in relation to current myalgic encephalomyelitis/chronic fatigue syndrome: **(A)** Multiplicative interaction analysis, **(B)** additive interaction analysis, DEPEV_A:0: no depression history, DEPEV_A:1: depression history, A0B0: lower score without depression history, A0B1: lower score with depression history, A1B0: higher score without depression history, A1B1: higher score with depression history).

Interaction analysis showed that the values of multiplicative scale, RERI, AP, and SI in all models (crude and model I) were not significant. The 95% CIs of RERI and AP included 0, and the 95% CIs of multiplicative scale and SI included 1, indicating that no significant multiplicative or additive interaction between disease history score and depression history was observed in relation to current ME/CFS (Supplementary Table 2). Figure 7 showed the multiplicative and additive interaction between disease history score and depression history in relation to current ME/CFS.

The disease history score and categorized score accounted for 45.3 and 19.3% of the association between depression history and current ME/CFS in the whole adults, respectively (model I, Supplementary Table 3). Disease history score accounted for 44.9% of the association between depression history and current ME/CFS in females (model I, Supplementary Table 3). Subgroup analyses in terms of age showed that disease history score accounted for 62.8% of the association between depression history and current ME/CFS in the 50–64 years group (model I, Supplementary Table 3).

**Figure 8:**
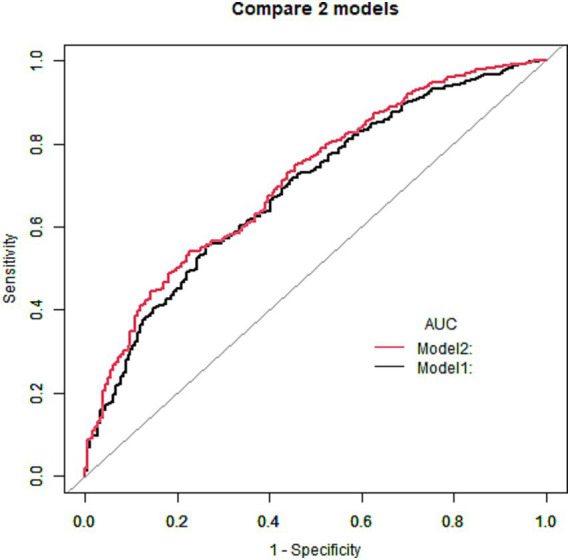
Comparison of AUC between two models for current myalgic encephalomyelitis/chronic fatigue syndrome of PSM data (model 1: Social, demographic + Dep model, model 2: Social, demographic + Dep + Score Model.

The sociodemographic characteristics + depression + disease history score model had a significantly higher AUC (0.7178 vs. 0.6933) compared with the sociodemographic characteristics + depression model (Supplementary Table 4; Figure 8). No significant differences in NRI and IDI between the sociodemographic characteristics + depression + disease history score model and the sociodemographic characteristics + depression model for identifying current ME/CFS were observed. These findings indicated that adding disease history score to the model did not produce a significantly better reclassification or discrimination improvement according to NRI and IDI (Supplementary Table 5).

**Figure 9:**
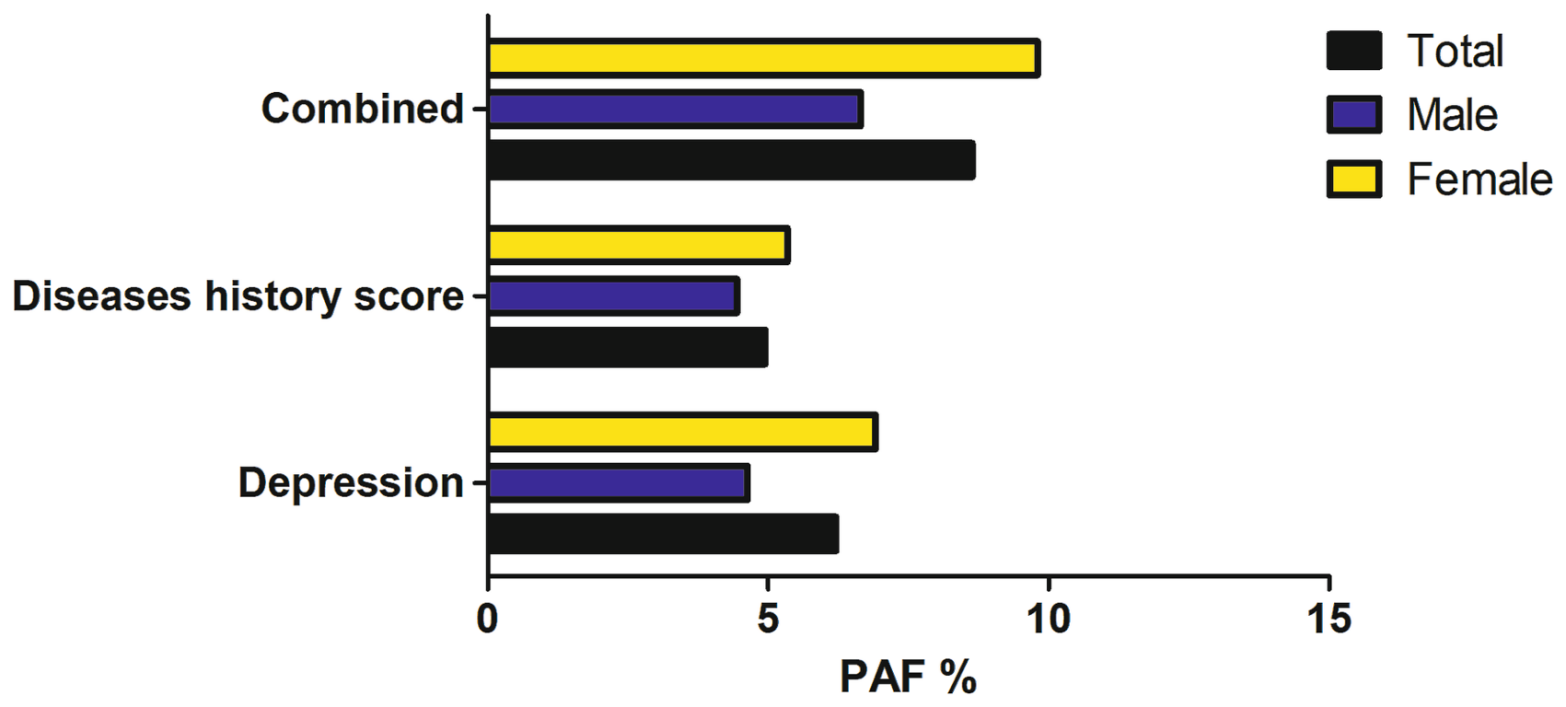
PAF of disease history score category and depression history for current myalgic encephalomyelitis/chronic fatigue syndrome of PSM data.

Supplementary Table 6 and Figure 9 presented the PAF of categorized disease history score and depression history for current ME/CFS. Depression history in females had a PAF of 6.91% for current ME/CFS. The combination of higher disease history score category and depression history showed a PAF of 9.80% for current ME/CFS in females. Depression history and higher disease history score category showed PAFs of 4.63 and 4.44% for current ME/CFS in males, respectively. These sensitivity analyses generally supported the robustness of the main association patterns.

## 4 Discussion

ME/CFS is an important public health burden across the world. Growing concern has been directed toward the adverse health impact of ME/CFS in the past decades. Given the unclear joint associations of depression history and multimorbid burden with current ME/CFS, this study focused on the independent and combined associations of depression history and the disease history score with current ME/CFS, as well as their interrelationships.

The study found that depression history was associated with 61% higher odds of current ME/CFS. Each unit increment in the disease history score above −2.03 was associated with 85% higher odds of current ME/CFS. The continuous and categorical disease history scores accounted for 47.2%/22.9% of the association between depression history and current ME/CFS in the overall population, and 44.4%/24.1% among females, respectively. The disease history score accounted for 59.2 and 30.5% of the depression-related association with current ME/CFS among participants aged 50–64 years and those aged 65 years and older. In females, the combined PAF of depression history and elevated disease history score reached 12.85%. Overall, depression history showed a stronger association with current ME/CFS than disease history score. Sensitivity analyses also yielded similar findings. However, because the study was cross-sectional, these findings do not establish whether depression preceded multimorbidity, whether multimorbidity preceded depression, or whether both developed before, after, or alongside ME/CFS.

Several plausible mechanisms may explain the findings of this study. First, many symptoms, such as sleep disturbances, poor concentration, and fatigue, tend to occur in both depression and CFS (15). Depressive mood and major depressive episodes are highly prevalent in CFS, while fatigue and somatic symptoms are common manifestations of depression (16). Depression and CFS share aberrations in inflammatory, oxidative, and nitrosative stress (17). Hypothalamic–pituitary–adrenal (HPA) axis dysfunction plays a role in the onset and maintenance of both CFS and depression (18). Shared immunoinflammatory pathways and activated microglia underpin the comorbid depression and CFS (19). Hence, depression may be associated with current ME/CFS through some overlapping biological and symptomatic pathways. Nevertheless, symptom overlap between depression, anxiety disorders, and ME/CFS may also inflate observed associations, especially for self-reported conditions. Fatigue, sleep disturbance, cognitive dysfunction, and somatic symptoms are shared across these conditions, making it difficult to distinguish true comorbidity from overlapping symptom reporting in cross-sectional survey data.

Second, chronic diseases are well-documented inducers of fatigue. For example, fatigue was one of the most frequently reported symptoms in diabetes. Depression and physical activity were significantly associated with fatigue in diabetes (20). Chronically fatigued diabetes mellitus 1 patients had more functional impairments than non-chronically fatigued patients (21). Fatigue is highly prevalent in rheumatoid arthritis (22). Fatigue is an important symptom after dyspnea in COPD (23). Fatigue is also a prevalent and disabling symptom in acute myocardial infarction and chronic heart failure (24). Mental and behavioral diseases yielded highest fatigue prevalence (25). Preexisting anxiety disorder led to an increased risk of CFS (11). The prevalence of atopy among CFS was about 80% (26). Based on the above-mentioned evidence, depression and fatigue may be common symptoms of many diseases in the later stage. Depressive state may deteriorate various disorders, which lead to the presence and exacerbation of fatigue. Therefore, the observed association between depression history and current ME/CFS may be partly explained by multimorbidity burden. However, the present study cannot determine whether multimorbidity lies on a causal pathway, acts as a confounder, or develops concurrently with depression and ME/CFS.

Third, fatigue is the most reported symptom in old age, and brain aging is closely associated with fatigue (27). Aging also modifies the immune response (28). Meanwhile, older adults with fatigue are often subject to deteriorating neurological, physiological, cognitive and emotional conditions (29).

In this sense, aging is associated with fatigue and chronic diseases. Sex disparity is another key consideration. Females have a higher risk of comorbid depression and anxiety, with a threefold higher prevalence of anxiety disorders compared with males (30). In contrast, males suffering from depression tend to be underdiagnosed (31). Women are likely to have a higher awareness of psychological and psychiatric disorders. Men are diagnosed with depression at half the rate of women (32). Therefore, depression and other disorders-related to ME/CFS are likely to be reported more frequently in females. In some common diseases, women also showed a comparatively poor outcome. For example, females with coronary heart diseases have worse outcomes than their male counterparts (33). Hypertension awareness is greater in women than men (34). Women tend to present with unstable angina or non-Q-wave infarction more frequently (35). These sex-based differences in mental health susceptibility, multimorbidity burden, disease prognosis, and health awareness may collectively explain the higher PAF of depression and disease history score for current ME/CFS in females.

No significant multiplicative or additive interaction was identified between depression history and the disease history score, indicating that depression history and multimorbidity burden showed largely independent associations with current ME/CFS. Meanwhile, no significant incremental benefits were observed in reclassification or discriminatory performance based on NRI and IDI. Although the AUC increased slightly after adding the disease history score, the nonsignificant NRI and IDI suggested that the disease history score provided limited incremental value for reclassifying individuals with current ME/CFS beyond sociodemographic factors and depression history. Therefore, the predictive implications of this score should be interpreted conservatively.

Of note, exploratory indirect association analyses showed that a considerable proportion of the depression-current ME/CFS association was accounted for by increased multimorbidity burden. These findings suggest a potential interrelationship among depression history, multimorbidity burden, and current ME/CFS, but they should not be interpreted as evidence of causal mediation.

In cross-sectional NHIS data, depression history, disease histories, ME/CFS history, and current ME/CFS status were measured in the same survey context, preventing confirmation of temporal sequence.

ME/CFS is a heterogeneous, multifactorial disorder driven by multiple pathogenic factors, including virus infection, immune dysregulation, neuroendocrine dysfunction, and oxidative stress (36). On the other hand, gut microbiome was also reported to be associated with fatigue symptoms in ME/CFS (37). Chemical toxins and psychological disturbances were risk factors for ME/CFS development (38). A recent retrospective cohort study further reported an increased risk of ME/CFS after COVID-19 infection, highlighting the potential role of infection-related triggers in ME/CFS research (39). Compared with that longitudinal electronic health record study, the present NHIS analysis was cross-sectional and self-reported; therefore, the findings should be interpreted as associations rather than incident risk estimates. Hence, ME/CFS represents the cumulative consequences of multifactorial insults. Investigating the correlates of current ME/CFS is essential for improving clinical characterization of long-term fatigue. Future studies should incorporate more biological indicators to identify additional mechanisms underlying current ME/CFS.

Nevertheless, this study had several strengths. First, the analyses between various disorders and current ME/CFS can reflect the association of multimorbidity with current ME/CFS status, which is important for understanding the clinical profile of adults with ME/CFS from the perspective of disease history. Meanwhile, identification of the threshold point of disease history score in relation to current ME/CFS can effectively show the dose–response association between cumulative multimorbidity burden and current ME/CFS. Second, comprehensive interaction and exploratory indirect association analyses clarified the independent versus indirect associations of depression history and disease history score, while ROC, NRI, and IDI analyses quantified their incremental discriminatory value. Third, PAF analysis indicated the association-based contribution of depression history and disease history score to current ME/CFS, providing evidence for identifying adults with depression history, multimorbidity burden, and current ME/CFS.

There were also some limitations which merited attention. First, this investigation was a cross- sectional study based on self-reported NHIS data. The recall or reporting bias may be inevitable. Depression history, disease histories, ME/CFS history, and current ME/CFS status were measured in the same survey context; therefore, temporal relationships could not be established. Reverse causation is possible, as ME/CFS may contribute to depressive symptoms, healthcare-seeking behavior, and reporting of other disease histories. Accordingly, the indirect association analyses should not be interpreted as causal mediation. Second, the final analytic sample was limited to adults with available ME/CFS history, current ME/CFS status, disease history variables, and complete covariates. The complete-case restriction and the large reduction from the overall NHIS sample may introduce selection bias and may affect representativeness. Future studies should compare included and excluded participants and consider multiple imputation or other missing-data methods when appropriate. Third, the disease history score was derived from LASSO coefficients optimized for current ME/CFS, which may raise concerns about circularity if the score is interpreted as an independent multimorbidity burden construct. This study therefore interpreted the score as an outcome-informed disease history index and provided the retained variables, coefficients, and formula to improve transparency and reproducibility. External validation or independent testing datasets are needed to evaluate the generalizability of the score. Fourth, important potential confounders were unavailable, including antidepressant use, sleep disorders, physical activity, pain severity, COVID-19 history, disease duration, healthcare utilization, and detailed mental health severity. Their absence may result in residual confounding. Finally, the lack of laboratory data may fail to fully reflect the physiological status. Incorporating laboratory inflammatory, endocrine, immune, and metabolic markers in future longitudinal studies will help further clarify the biological mechanisms underlying current ME/CFS.

## 5 Conclusion

Taken together, the results of this study indicated that the disease history score may partly explain the association between depression history and current ME/CFS among US adults, particularly among females and adults aged 50 years or older. Depression history showed a stronger association with current ME/CFS than increased disease history score category. These findings support the potential relevance of depression history and multimorbidity burden in identifying adults with current ME/CFS, while longitudinal studies are needed to clarify temporal relationships.

## Data Availability

The original contributions presented in the study are included in the article/Supplementary material, further inquiries can be directed to the corresponding author.
