# Supplementary material for: The role of disease history score in the association between depression history and current ME/CFS among US adults
Source: Front Public Health. 2026 Jul 21;14:1862602. doi: 10.3389/fpubh.2026.1862602 (PMC13433158; doi:10.3389/fpubh.2026.1862602)
Supplement: Supplementary file 1 [file Table_1.DOC]

**eTable 1 Baseline characteristics of adult participants before and after PSM**

Characteristics	Participants before PSM	Participants after PSM

Non-depression	depression	SMD	Non-depression	depression	SMD

(487)	(813)	(437)	(437)

| Age, Mean±SD | 61.75±14.51 | 55.27±15.51 | -0.417 | 60.66±14.60 | 60.68±14.42 | 0.0013 |
| --- | --- | --- | --- | --- | --- | --- |
| Sex, n (%) |  |  |  |  |  |  |
| Female | 303 (62.22) | 584 (71.83) | 0.214 | 279 (63.8) | 290 (66.4) | 0.0528 |
| Male | 184 (37.78) | 229 (28.17) | -0.214 | 158 (36.2) | 147 (33.6) | -0.0528 |
| Household region,  n (%) |  |  |  |  |  |  |
| Northeast | 86(17.66) | 108(13.28) | -0.129 | 78 (17.8) | 59 (13.5) | -0.1198 |
| Midwest | 89(18.28) | 188 (23.12) | 0.115 | 81 (18.5) | 104 (23.8) | 0.1291 |
| South | 202 (41.48) | 340 (41.82) | 0.007 | 175 (40) | 178 (40.7) | 0.0140 |
| West | 110 (22.59) | 177 (21.77) | -0.020 | 103 (23.6) | 96 (22) | -0.0382 |
| District, n (%) |  |  |  |  |  |  |
| Nonmetropolitan | 93(19.10) | 183(22.51) | 0.082 | 85 (19.5) | 87 (19.9) | 0.0115 |
| Medium and  small metro | 172(35.32) | 299(36.78) | 0.030 | 159 (36.4) | 162 (37.1) | 0.0142 |
| Large fringe metro | 103(21.25) | 147(18.08) | -0.080 | 87 (19.9) | 81 (18.5) | -0.0348 |
| Large central metro | 119 (24.44) | 184 (22.63) | -0.043 | 106 (24.3) | 107 (24.5) | 0.0053 |
| Race, n (%) |  |  |  |  |  |  |
| White only | 414 (85.01) | 679 (83.52) | -0.040 | 371 (84.9) | 368 (84.2) | -0.0190 |
| African  American only | 42 (8.62) | 76 (9.35) | 0.025 | 38 (8.7) | 43 (9.8) | 0.0395 |
| Asian only | 13 (2.67) | 13 (1.60) | -0.085 | 11 (2.5) | 11 (2.5) | 0.0000 |
| AIAN only | 7 (1.44) | 16 (1.97) | 0.038 | 7 (1.6) | 6 (1.4) | -0.0189 |
| AIAN and any  other group | 9 (1.85) | 18 (2.21) | 0.025 | 8 (1.8) | 7 (1.6) | -0.0176 |
| Other single  and multiple races | 2 (0.41) | 11 (1.35) | 0.082 | 2 (0.5) | 2 (0.5) | 0.0000 |
| Educational level  , n (%) |  |  |  |  |  |  |
| Grade 1 to 11 | 32 (6.57) | 55 (6.77) | 0.008 | 32 (7.3) | 26 (5.9) | -0.0552 |
| 12th grade  no diploma | 8 (1.64) | 20 (2.46) | 0.053 | 8 (1.8) | 10 (2.3) | 0.0322 |
| GED or equivalent | 19 (3.90) | 37 (4.55) | 0.031 | 17 (3.9) | 21 (4.8) | 0.0449 |
| High  School Graduate | 120 (24.64) | 189 (23.25) | -0.033 | 106 (24.3) | 106 (24.3) | 0.000 |
| Some colleges  no degree | 88 (18.07) | 184 (22.63) | 0.109 | 81 (18.5) | 91 (20.8) | 0.0576 |
| Associate degree:  occupational technical or  vocational program | 19 (3.90) | 42 (5.17) | 0.057 | 18 (4.1) | 26 (5.9) | 0.0838 |
| Associate degree: academic program | 63 (12.94) | 92 (11.32) | -0.051 | 56 (12.8) | 43 (9.8) | -0.0940 |
| Bachelor’s degree | 82 (16.84) | 123 (15.13) | -0.048 | 71 (16.2) | 77 (17.6) | 0.0366 |
| Master’s degree | 37 (7.60) | 53 (6.52) | -0.044 | 31 (7.1) | 27 (6.2) | -0.0368 |
| Professional School  or Doctoral degree | 19 (3.90) | 18 (2.21) | -0.115 | 17 (3.9) | 10 (2.3) | -0.0927 |
| BMI category  , n (%) |  |  |  |  |  |  |
| Underweight | 10 (2.05) | 20 (2.46) | 0.026 | 9 (2.1) | 11 (2.5) | 0.0306 |
| Healthy weight | 137 (28.13) | 204 (25.09) | -0.070 | 119 (27.2) | 114 (26.1) | -0.0259 |
| Overweight | 141 (28.95) | 225 (27.68) | -0.029 | 125 (28.6) | 133 (30.4) | 0.0401 |
| Obese | 199 (40.86) | 364 (44.77) | 0.079 | 184 (42.1) | 179 (41) | -0.0232 |
| Smoking status  , n (%) |  |  |  |  |  |  |
| Never smoker | 250 (51.33) | 335 (41.21) | -0.206 | 216 (49.4) | 209 (47.8) | -0.0321 |
| Former smoker | 164 (33.68) | 268 (32.96) | -0.015 | 148 (33.9) | 152 (34.8) | 0.0193 |
| Current some  day smoker | 13 (2.67) | 43 (5.29) | 0.117 | 13 (3) | 15 (3.4) | 0.0260 |
| Current every  day smoker | 60 (12.32) | 167 (20.54) | 0.203 | 60 (13.7) | 61 (14) | 0.0066 |
| Family poverty  ratio, Mean ± SD | 3.52±2.79 | 2.64±2.31 | -0.382 | 3.29±2.62 | 3.12±2.63 | -0.0646 |

PSM: propensity score matching, AIAN: American Indian/Alaska Native, GED: general educational development.

**eTable 2 Interaction between depression history and disease history score category in relation to current ME/CFS**

Group	Crude	Model I

OR (95% CI) P	OR（95% CI） P

| Lower disease history score (< median) |  |  |
| --- | --- | --- |
| without depression | Reference | Reference |
| with depression | 1.65 (1.05, 2.60) | 1.64 (1.02, 2.64) |
| Higher disease history score (≥ median) |  |  |
| without depression | 1.70 (1.03, 2.80) | 1.44 (0.84, 2.48) |
| with depression | 3.02 (1.95, 4.68) | 2.68 (1.70, 4.24) |
| Multiplicative scale | 1.08 (0.52, 2.24) | 1.05 (0.49, 2.24) |
| RERI | 0.68 (-0.79, 2.15) | 0.49 (-0.92, 1.91) |
| AP | 0.22 (-0.21, 0.66) | 0.18 (-0.3, 0.66) |
| SI | 1.5 (0.59, 3.84) | 1.4 (0.5, 3.89) |

ME/CFS: myalgic encephalomyelitis/chronic fatigue syndrome, OR: odds ratio, RERI: relative excess risk due to interaction, AP: attributable proportion

due to interaction, SI: synergy index, Crude: no adjust Model I: adjust for age, BMI category, educational level, poverty ratio, race, region, sex,

smoking status, and district.

**eTable 3 Exploratory indirect association analysis of disease history score in the depression-current ME/CFS association**

Exposure	Crude	Model I

β/OR (95% CI) P	β/OR(95% CI）P

| Score |  |  |
| --- | --- | --- |
| Total effect | 0.113 (0.064, 0.171) <0.0001 | 0.109 (0.0613, 0.166) <0.0001 |
| Direct effect | 0.057 (0.008, 0.118) 0.0240 | 0.0597 (0.0117, 0.121) 0.0220 |
| Mediation effect | 0.056 (0.029, 0.081) <0.0001 | 0.0494 (0.0227, 0.0719) <0.0001 |
| Proportion mediated effect | 0.498 (0.245, 0.871) <0.0001 | 0.453 (0.186, 0.829) <0.0001 |
| Score category |  |  |
| Total effect | 0.113 (0.0624, 0.167) <0.0001 | 0.107 (0.0583, 0.159) <0.0001 |
| Direct effect | 0.085 (0.031, 0.138) 0.0020 | 0.087 (0.034, 0.139) <0.0001 |
| Mediation effect | 0.028 (0.011, 0.050) <0.0001 | 0.0207 (0.002, 0.0412) 0.0260 |
| Proportion mediated effect | 0.250 (0.088, 0.552) <0.0001 | 0.193 (0.018, 0.489) 0.0260 |
| Female |  |  |
| Score |  |  |
| Total effect | 0.118 (0.048, 0.185) 0.002 | 0.119 (0.049, 0.184) <0.0001 |
| Direct effect | 0.064 (-0.009, 0.137) 0.072 | 0.066 (-0.009, 0.130) 0.078 |
| Mediation effect | 0.054 (0.017, 0.083) <0.0001 | 0.053 (0.026, 0.085) <0.0001 |
| Proportion mediated effect | 0.456 (0.141, 1.143) 0.002 | 0.449 (0.205, 1.160) <0.0001 |
| Score category |  |  |
| Total effect | 0.125 (0.0559, 0.184) <0.0001 | 0.121 (0.050, 0.188) <0.0001 |
| Direct effect | 0.096 (0.026, 0.161) 0.004 | 0.098 (0.0266, 0.170) <0.0001 |
| Mediation effect | 0.0289 (0.0058, 0.053) 0.004 | 0.022 (-0.0001, 0.0444) 0.0540 |
| Proportion mediated effect | 0.232 (0.047, 0.593) 0.004 | 0.185 (-0.003, 0.5455) 0.0540 |
| Male |  |  |
| Score |  |  |
| Total effect | 0.093 (-0.005, 0.186) 0.060 | 0.087 (-0.007, 0.172) 0.070 |
| Direct effect | 0.0357 (-0.064, 0.134) 0.490 | 0.0476 (-0.0533, 0.143) 0.382 |
| Mediation effect | 0.057 (0.017, 0.1027) 0.004 | 0.0396 (-0.003, 0.0844) 0.0760 |
| Proportion mediated effect | 0.616 (-1.100, 3.127) 0.0640 | 0.454 (-0.718, 3.646) 0.142 |
| Score category |  |  |
| Total effect | 0.093 (-0.003, 0.185) 0.056 | 0.0889 (-0.007, 0.169) 0.0720 |
| Direct effect | 0.064 (-0.041, 0.162) 0.232 | 0.0663 (-0.0372, 0.159) 0.208 |
| Mediation effect | 0.0287 (-0.006, 0.0698) 0.112 | 0.0226 (-0.0169, 0.0619) 0.314 |
| Proportion mediated effect | 0.309 (-0.420, 2.251) 0.168 | 0.254 (-0.832, 2.297) 0.362 |
| 18-34 years |  |  |
| Score |  |  |
| Total effect | -0.0714 (-0.3136, 0.0954) 0.356 | NA |
| Direct effect | -0.2156 (-0.4204, -0.0661) 0.014 | NA |
| Mediation effect | 0.1441 (0.0248, 0.3148) 0.008 | NA |
| Proportion mediated effect | -2.0182 (-20.7272, 24.6693) 0.364 | NA |
| Score category |  |  |
| Total effect | -0.051 (-0.304, 0.169) 0.580 | -0.017 (-0.408, 0.333) 0.824 |
| Direct effect | -0.092 (-0.326, 0.133) 0.396 | -0.082 (-0.452, 0.347) 0.652 |
| Mediation effect | 0.041 (-0.026, 0.115) 0.260 | 0.065 (-0.112, 0.241) 0.422 |
| Proportion mediated effect | -0.806 (-7.630, 4.238) NA | -3.756 (-7.859, 4.476) 0.806 |
| 35-49 years |  |  |
| Score |  |  |
| Total effect | 0.221 (0.009, 0.412) 0.044 | 0.129 (-0.036, 0.285) 0.116 |
| Direct effect | 0.097 (-0.090, 0.252) 0.360 | 0.0888 (-0.089, 0.272) 0.276 |
| Mediation effect | 0.124 (0.045, 0.254) <0.0001 | 0.040 (-0.0479, 0.115) 0.340 |
| Proportion mediated effect | 0.562 (0.145, 2.062) 0.044 | 0.310 (-1.809, 2.153) 0.440 |
| Score category |  |  |
| Total effect | 0.213 (0.075, 0.359) 0.002 | 0.134 (-0.039, 0.298) 0.112 |
| Direct effect | 0.147 (0.010, 0.282) 0.042 | 0.124 (-0.064, 0.292) 0.162 |
| Mediation effect | 0.0655 (0.019, 0.146) 0.012 | 0.010 (-0.059, 0.073) 0.692 |
| Proportion mediated effect | 0.308 (0.088, 0.908) 0.014 | 0.075 (-0.882, 1.091) 0.752 |
| 50-64 years |  |  |
| Score |  |  |
| Total effect | 0.127 (0.044, 0.215) <0.0001 | 0.108 (0.017, 0.191) 0.010 |
| Direct effect | 0.0498 (-0.044, 0.149) 0.2780 | 0.040 (-0.0599, 0.142) 0.4280 |
| Mediation effect | 0.0773 (0.0284, 0.127) <0.0001 | 0.068 (0.020, 0.124) 0.006 |
| Proportion mediated effect | 0.608 (0.201, 1.731) <0.0001 | 0.628 (0.149, 2.937) 0.0160 |
| Score category |  |  |
| Total effect | 0.128 (0.043, 0.215) <0.0001 | 0.108 (0.012, 0.192) 0.020 |
| Direct effect | 0.089 (-0.008, 0.184) 0.062 | 0.078 (-0.023, 0.173) 0.140 |
| Mediation effect | 0.0387 (-0.004, 0.0788) 0.076 | 0.030 (-0.0097, 0.076) 0.146 |
| Proportion mediated effect | 0.304 (-0.027, 1.139) 0.076 | 0.282 (-0.141, 1.378) 0.166 |
| 65 years and over |  |  |
| Score |  |  |
| Total effect | 0.093 (0.011, 0.175) 0.028 | 0.092 (0.002, 0.173) 0.044 |
| Direct effect | 0.068 (-0.0166, 0.154) 0.106 | 0.066 (-0.022, 0.146) 0.156 |
| Mediation effect | 0.0247 (0.0023, 0.0512) 0.036 | 0.026 (0.0009, 0.0544) 0.0420 |
| Proportion mediated effect | 0.265 (-0.0122, 1.397) 0.060 | 0.281 (-0.0822, 1.538) 0.078 |
| Score category |  |  |
| Total effect | 0.092 (0.009, 0.176) 0.032 | 0.0922 (0.00178, 0.174) 0.044 |
| Direct effect | 0.082 (-0.005, 0.163) 0.066 | 0.0838 (-0.007, 0.165) 0.072 |
| Mediation effect | 0.010 (-0.012, 0.037) 0.2840 | 0.0085 (-0.017, 0.0355) 0.4520 |
| Proportion mediated effect | 0.109 (-0.231, 0.874) 0.304 | 0.0924 (-0.3607, 0.7343) 0.4760 |

ME/CFS: myalgic encephalomyelitis/chronic fatigue syndrome, OR: odds ratio, Crude: no adjust Model I: adjust for age, BMI category, educational

level, poverty ratio, race, region, sex, smoking status, and district.

**eTable 4 Discriminatory performance of different models for current ME/CFS**

Models	AUC	95% CI	Accuracy	Sensitivity	Specificity	PPV	NPV	P

| ^1^Social, demographic | 0.6765 | 0.6358-  0.7192 | 0.5824 | 0.5520 | 0.6978 | 0.8741 | 0.2906 |  |
| --- | --- | --- | --- | --- | --- | --- | --- | --- |
| ^2^Social, demographic + Dep | 0.6933 | 0.6505-  0.7298 | 0.5927 | 0.5549 | 0.7363 | 0.8889 | 0.3032 | 0.1245  2 vs 1 |
| ^3^Social,  demographic+Dep+Score | 0.7178 | 0.6755-  0.7592 | 0.5904 | 0.5419 | 0.7747 | 0.9014 | 0.3079 | 0.0191  3 vs 2 |

ROC: receiver operating curve, ME/CFS: myalgic encephalomyelitis/chronic fatigue syndrome, Dep: depression, PPV: positive predictive value, NPV:

negative predictive value.

**eTable 5 NRI and IDI for the analysis of different models for current ME/CFS (Social, demographic +Dep vs**

**Social, demographic+Dep+Score)**

Index	Estimate	P value	95% Lower	95% Upper

| NRI | 0.0255 | 0.4061 | -0.0346 | 0.0855 |
| --- | --- | --- | --- | --- |
| IDI | 0.0255 | 0.4071 | -0.0347 | 0.0856 |

ME/CFS: myalgic encephalomyelitis/chronic fatigue syndrome, Dep: depression, NRI: net reclassification index, IDI: integrated discrimination index.

**eTable 6 PAF of disease history score category and depression history for current ME/CFS**

Exposure	PAF, % (95% CI)

| Total |  |
| --- | --- |
| Depression | 6.21 (2.91-9.51) |
| Disease history scores category | 4.95 (1.94-7.97) |
| Disease history scores category + Depression | 8.63 (4.49-12.78) |
| Female |  |
| Depression | 6.91 (2.83-10.99) |
| Disease history scores category | 5.34(1.45-9.22) |
| Disease history scores category + Depression | 9.80 (4.36-15.24) |
| Male |  |
| Depression | 4.63 (-1.04-10.31) |
| Disease history scores category | 4.44 (-0.79-9.68) |
| Disease history scores category + Depression | 6.64 (-0.24-13.52) |

PAF: population attributable fraction, OR: odds ratio, CI: confidence intervals. adjust for district, race, smoking, BMI category, educational level, region,

sex, age category, and poverty ratio category.
